# Evaluation of a Pediatric Obesity Management Toolkit for Health Care Professionals: A Quasi-Experimental Study

**DOI:** 10.3390/ijerph18147568

**Published:** 2021-07-16

**Authors:** Barkha P. Patel, Stasia Hadjiyannakis, Laurie Clark, Annick Buchholz, Rebecca Noseworthy, Julie Bernard-Genest, Catharine M. Walsh, Amy C. McPherson, Jonah Strub, Michele Strom, Jill K. Hamilton

**Affiliations:** 1 Division of Endocrinology, The Hospital for Sick Children, Toronto, ON M5G 1X8, Canada; barkha.patel@sickkids.ca (B.P.P.); rebecca.noseworthy@sickkids.ca (R.N.); jonah.strub@gmail.com (J.S.); michelestrom@hotmail.com (M.S.); 2 Children’s Hospital of Eastern Ontario Research Institute, Ottawa, ON K1H 5B2, Canada; shadjiyannakis@cheo.on.ca; 3 Department of Pediatrics, University of Ottawa, Ottawa, ON K1N 6N5, Canada; 4 Centre for Healthy Active Living, Children’s Hospital of Eastern Ontario, Ottawa, ON K1H 8L1, Canada; laclark@cheo.on.ca (L.C.); abuchholz@cheo.on.ca (A.B.); 5 Centre Mère-enfant Soleil du Centre Hospitalier Universitaire de Québec, Université Laval, Québec City, QC G1V 0A6, Canada; julie.bernard-genest.1@ulaval.ca; 6 Division of Gastroenterology, Hepatology and Nutrition and the Research and Learning Institutes, The Hospital for Sick Children, Toronto, ON M5G 1X8, Canada; catharine.walsh@sickkids.ca; 7 Department of Paediatrics and the Wilson Centre, University of Toronto, Toronto, ON M5S 1A4, Canada; 8 Bloorview Research Institute, Holland Bloorview Kids Rehabilitation Hospital, Toronto, ON M4G 1R8, Canada; amcpherson@hollandbloorview.ca; 9 Department of Nutritional Sciences, University of Toronto, Toronto, ON M5S 1A4, Canada

**Keywords:** obesity, self-efficacy, pediatrics, healthcare professionals, videos

## Abstract

Health care professionals (HCPs) play a critical role in helping to address weight-related issues with pediatric patients, yet often feel ill-equipped to discuss/manage this complex and sensitive health issue. Using the five As (“Ask, Assess, Advise, Agree, and Assist”) of Pediatric Obesity Management, we created a series of educational videos and evaluated the content, quality (acceptability, engagement), and impact of these videos on HCPs’ self-efficacy, knowledge, and change in practice when addressing weight-related issues with pediatric patients and their families using questionnaires. HCPs (*n* = 65) participated in a baseline assessment and 4–6 month follow-up (*n* = 54). Knowledge and self-efficacy increased post-video for the majority of participants. At follow-up, most HCPs reported a change in their practice attributable to viewing the videos, and their self-efficacy ratings improved over time for the majority of questions asked. Most participants rated aspects of each of the videos highly. Preliminary findings suggest that an evidence-based educational toolkit of videos, based on the 5As framework, may lead to changes in self-reported behaviors among HCPs, and sustained improvements in their self-efficacy in addressing weight-related topics with children and their families. (Clinical Trial Number NCT04126291).

## 1. Introduction

Children and adolescents with obesity encounter significant short- and long-term complications that may impact their physical health and quality of life as adults [1]. There is an urgent need to prevent and effectively manage childhood obesity and related co-morbidities as early in life as possible. Health care professionals (HCPs) play a critical role in helping to address weight-related issues with patients and their families [2]. At the same time, HCPs often feel ill-equipped to manage this complex and sensitive health issue in children and youth, in particular [3,4]. Specifically, pediatricians and primary care physicians have self-reported that they feel unprepared to adequately provide care to children with obesity [5,6,7]. Barriers to discussing weight during appointments with patients include a lack of self-efficacy, inadequate training, fear of damaging their relationship with patients and their families, and fear of triggering other issues such as eating disorders [3,8,9,10]. Parents and children with obesity have described experiences of poor communication, shame, and blame when interacting with HCPs [11,12].

Obesity-specific education is lacking within the medical school curriculum and as a component of residencies and fellowship programs [13,14]. Therefore, evidence-based resources may help improve provider–client interactions by outlining strategies for HCPs to use in supporting their patients in obesity prevention and management [15,16]. HCPs benefit from engaging in medical education, which often comprises multimedia learning [17]. Research has shown that educational videos are an effective means to improve knowledge, confidence, and attitudes in this population [18,19,20,21]. Educational videos alongside a lecture series and reading material that focused on bias, diagnosis, and management of pediatric obesity decreased anti-obesity bias in pediatric residents [22]. It remains unclear, however, whether videos discussing how to manage weight with pediatric patients and their families leads to behavior change in HCPs.

“The 5As of Adult Obesity Management” is a recognized framework for increasing behavior change [23], and improving communication amongst providers and patients in weight management [24]. Published by Obesity Canada, the 5As stands for “Ask, Assess, Advise, Agree, and Assist.” In 2013, this tool was adapted as a booklet for the pediatric population [25]. Although this resource is a helpful tool for HCPs, feedback from focus groups indicate that HCPs would like an expanded version with practical visuals of how a 5As framed encounter would occur in a busy office setting. Using best practices in education pedagogy and multimedia development principles, we previously developed an educational whiteboard video for HCPs outlining strategies to communicate about weight with children and their families, based on the first A (“Ask”) [26]. We found that HCPs’ self-reported self-efficacy for initiating conversations with patients with low and high body weights significantly improved immediately following the video, with no significant decline 4–6 months later, indicating skill retention [26].

Based on our previous study using video-based education, as well as recent evidence that greater obesity training increases physician confidence [27], we predicted that expanding our educational resources to create a series of evidence-based videos (a “toolkit”) discussing how to comprehensively use the 5As approach, would improve knowledge of how to address weight-related issues with patients and their families and self-efficacy in HCPs.

Therefore, the objectives of the current study were to evaluate the content, quality (acceptability, engagement), and impact of these videos on HCPs’ self-efficacy, knowledge, and change in practice when addressing weight-related issues with pediatric patients and their families using the 5As framework.

## 2. Materials and Methods

### 2.1. Video Development

To determine evidence-based best practices for communicating about weight, we conducted a comprehensive scoping review [28], as well as focus groups with parents and youth to investigate their perceptions of how HCPs should address weight-related topics in relation to the extant literature [11]. As a result of this work, key messages and themes for the video content were identified, as well as the information in the 5As and the Edmonton Obesity Staging System for Pediatrics [29]. For the style of video, whiteboard animation was selected, which depicts hand-drawn, simple visuals on a white background to help explain complex information in an interactive manner. Six 8-min, high-definition, animated whiteboard videos were developed using Mayer’s principles of multimedia design, which have been shown to enhance learning [17]. The videos were created using VideoScribe (Version 3.2.1 PRO, Sparkol Inc., Brooklyn, NY, USA) and Graphic software (Version 3.1). This toolkit of videos can be viewed at https://meant2prevent.ca/5as-of-pediatric-obesity-management/ (accessed on 21 August 2020) and are briefly described in Table 1.

Twenty different HCPs and six parents of patients participated in usability testing of the videos (ten HCPs and three parents per cycle). Patients attended the obesity clinic at The Hospital for Sick Children (SickKids), Toronto, Canada. A research team member interviewed each HCP and parent following a viewing of the video. Participants were asked questions about content, video length, presentation of information, and satisfaction upon viewing. Comments concerning what was most and least useful, what could be done differently, and suggestions for improvement were identified. Following the first cycle, content analysis of the interviews, field notes, and the questionnaires helped to refine each video based on common themes. A second iterative cycle of usability testing was then conducted to identify additional recommendations for change. After the two cycles, the videos were then refined for use in this study.

### 2.2. Participants/Data Collection

This quasi-experimental design used a pre- and post-video evaluation, similar to our previous study [26], and a 4–6 month follow-up to assess if self-efficacy scores would be maintained in the short-term. The study was conducted at SickKids hospital in Toronto, Canada. This study was submitted and approved by the SickKids Institutional Research Ethics Board (#1000060439). The study was performed in accordance with the ethical standards as laid down in the 1964 Declaration of Helsinki.

We targeted HCPs who provide primary care to children and adolescents through SickKids, as well as hospitals, universities, and practices in the Greater Toronto Area. Participants included dietitians, nurses, nurse practitioners, and physicians, as well as trainees from these professions who work with youth. Posters were displayed at SickKids, and emails were distributed to relevant departments in the hospital, and throughout the city network of community pediatricians and associated allied health professionals. Participants who were interested contacted the research team directly through email or by phone. They were emailed a letter detailing the purpose of the study and a secure internet link to access the videos and pre/post questionnaires using the Research Electronic Data Capture (REDCap) portal [30]. Participants provided informed consent by completing and submitting the questionnaires. 

Participants first completed a pre-questionnaire on demographics, information about their current practice (i.e., how often they saw children who had overweight or obesity, as well as familiarity with using the 5As), and ratings of perceived knowledge and self-efficacy for a number of topics related to weight science, body mass index (BMI), and managing obesity. Refer to Supplementary files 1 and 2 for a copy of the questionnaires. Knowledge and self-efficacy around pediatric weight management were assessed by having participants rate themselves on a scale from 0 to 100. Questions on self-efficacy or how confident HCPs felt performing a variety of tasks were built according to the Bandura guide for constructing self-efficacy scales [31]. Participants were then prompted to watch the first video. Immediately after the video, they completed a satisfaction questionnaire, and rated their perceived change in knowledge and self-efficacy on a 7-point Likert scale as “No change (or reduction in knowledge)”, “Almost the same”, “A little better”, “Somewhat better”, “Moderately better”, “Better”, or “A great deal better”. They were then prompted to watch the next video and so forth, until they watched all 6 videos and completed all post-video questionnaires. Participants were permitted to stop and start the videos at any time to avoid fatigue. If participants agreed to be contacted after 4–6 months, they were emailed a short questionnaire to rate their satisfaction, self-efficacy, and perceived impact on practice. The questionnaires in the current study were adapted from a study using a questionnaire to evaluate an educational website for teenagers [32]. Within the questionnaire, an open-ended response section allowed participants to provide any additional comments or feedback.

### 2.3. Study Outcomes

The primary outcome measure was the change in knowledge and self-efficacy related to pediatric obesity management between baseline and immediately after watching the video. The secondary outcome measures were the change in self-efficacy related to pediatric weight management between baseline and follow-up (4–6 months), and the change in practice related to pediatric weight management at follow-up.

### 2.4. Statistical Analysis

Statistical Analysis Software (SAS) version 9.4 (SAS Institute Inc., Carey, NC, USA) was used for this study. Data are presented as means ± SEM, unless otherwise indicated. Significance was considered at *p* < 0.05. Descriptive statistics of baseline HCP demographic and practice variables were presented as percentages or median and interquartile ranges where applicable. For the follow-up analysis, the effects of time (baseline and 4–6 month follow-up) on self-efficacy ratings were analyzed using a PROC MIXED procedure for normally distributed data or a PROC GLIMMIX procedure for non-normal data. Knowledge and self-efficacy ratings were adjusted for sex, ethnicity, number of years in practice, and whether the 5As were used in practice. Post-hoc analysis by the Tukey–Kramer test was performed when main and interaction effects were found to be statistically significant. A sample size of 51 participants achieves 80% power to detect a mean change of 10 points on the self-efficacy scale between pre- and 4-to-6 months later with an estimated standard deviation of 25 and α of 0.05. We recruited 65 participants to account for loss to follow up.

## 3. Results

### 3.1. Participant Characteristics

**Figure 1:**
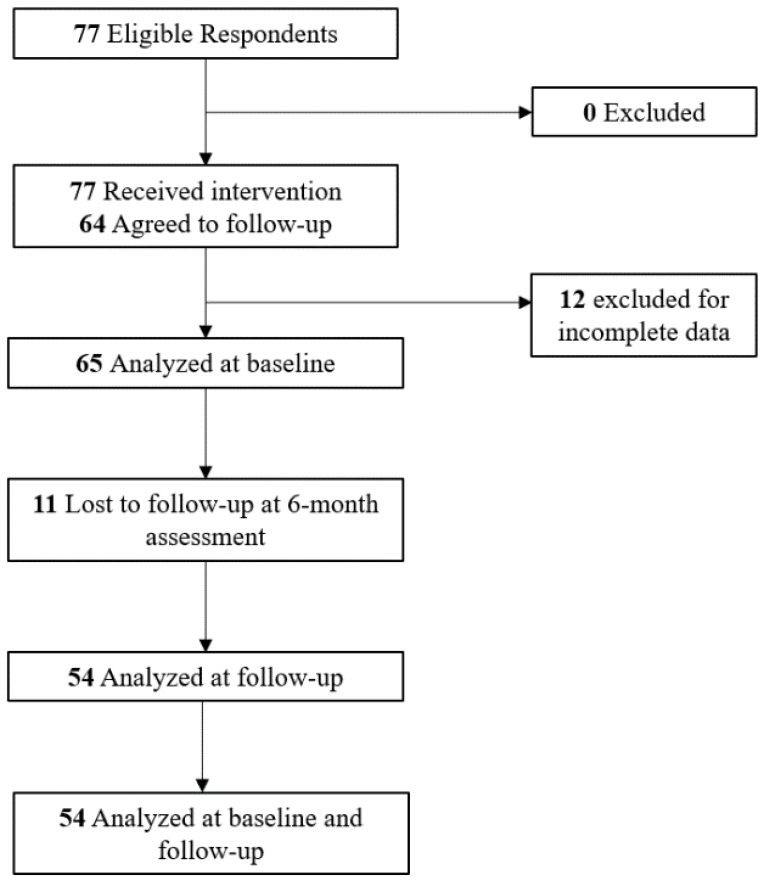
Consolidated Standards for Reporting of Trials (CONSORT) flow diagram.

Sixty-five HCPs completed the baseline questionnaire and the post-video responses (Figure 1). In regards to profession, 98.5% were registered healthcare professionals and 1.5% were trainees (residents/fellows). There was an equal percentage of general pediatric physicians and registered dietitians (31.3%), followed by nurse or nurse practitioners (28.1%), and finally, family physicians and physicians with sub-specialties (4.7%). Participant characteristics are presented in Table 2. In brief, the majority of participants were white females between the ages of 25–34 years. The median number of years in practice was 9.5 [2.9–20.0]. 

### 3.2. Questions about Current Practice and 5A’s

In our sample (Table 3), the majority (36.9%) saw at least one patient a week, did not use the 5As Approach to Pediatric Obesity Management (55.4%), and had not received training on using the 5As (78.5%). For those who received training, 54.5% reported doing so at a professional academic/education meeting, using a combination of training techniques (36.4%). The majority of training was <1 h (54.5%).

### 3.3. Baseline and Post-Video Knowledge and Self-Efficacy Ratings

**Figure 2:**
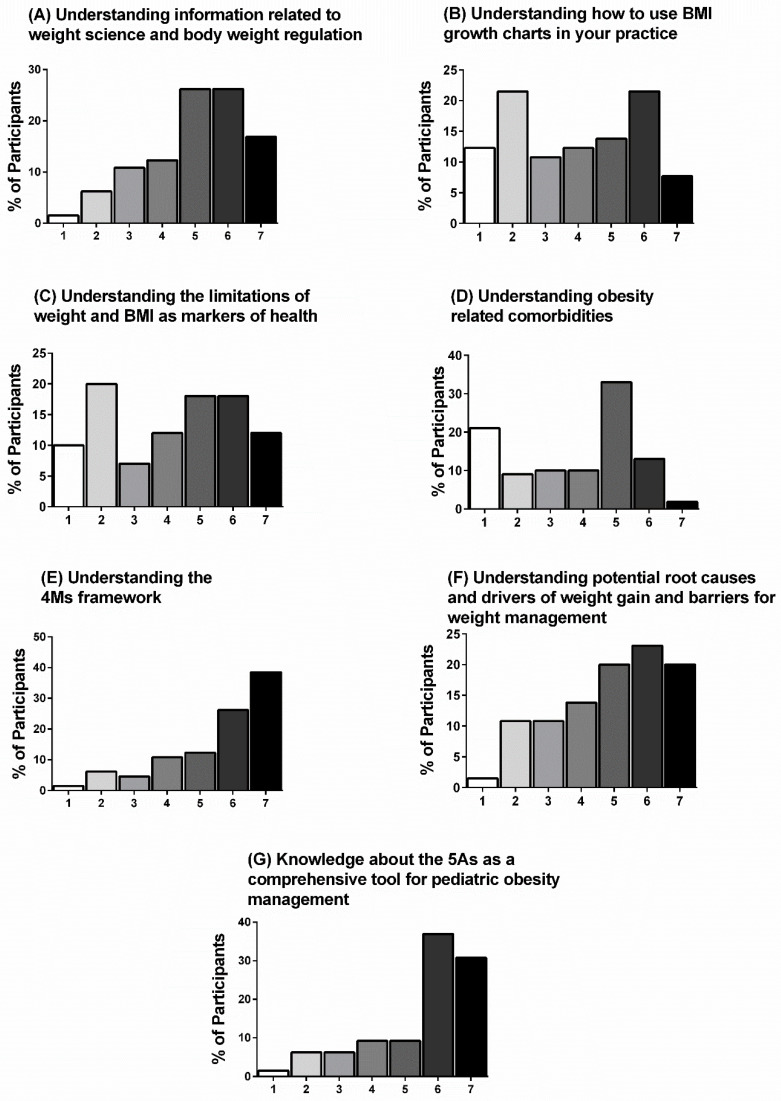
(**A**–**G**) Post-video change in knowledge. Participant agreement (%) measured using a Likert-type scale (1 to 7, where 1 = No change (or reduction in knowledge), 2 = Almost the same, 3 = A little better, 4 = Somewhat better, 5 = Moderately better, 6 = Better, and 7 = A great deal better) is shown for each statement (*n* = 65).

Baseline knowledge ratings are presented for each question in Table 4 and ratings or perceived change in knowledge are presented in Figure 2A–G. Baseline ratings were the lowest for “understanding the 4Ms framework” and “knowledge about the 5As as a comprehensive tool for pediatric obesity management”, while 77% of participants ranked their post-video change in knowledge for these questions as “moderately better” to “a great deal better.” Conversely, baseline ratings were the highest for “understanding how to use BMI growth charts in your practice” and “understanding the limitations of weight and BMI as markers of health”, while the majority of participants (49–57%) ranked their post-video change in knowledge for these questions as “no change” to “somewhat better.”

**Figure 3:**
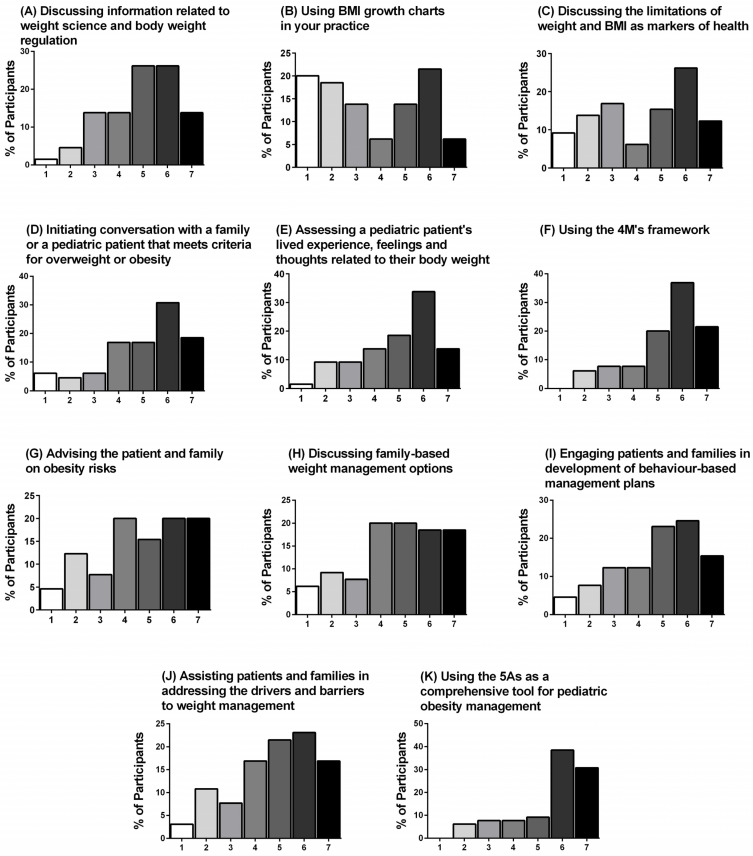
(**A**–**K**) Post-video change in self-efficacy. Participant agreement (%) measured using a Likert-type scale (1 to 7, where 1 = No change (or reduction in self-efficacy), 2 = Almost the same, 3 = A little better, 4 = Somewhat better, 5 = Moderately better, 6 = Better, and 7 = A great deal better) is shown for each statement (*n* = 65).

Baseline self-efficacy/confidence ratings are presented for each question in Table 5 and ratings of perceived change in self-efficacy are presented in Figure 3A–K. Consistent with the knowledge ratings, baseline ratings were the lowest for “using the framework” and “using the 5As as a comprehensive tool for pediatric obesity management”, while 78% of participants ranked their post-video change in self-efficacy for these questions as “moderately better” to “a great deal better.” Conversely, baseline ratings were the highest for “using BMI growth charts in your practice”, and the post-video change in self-efficacy was ranked as “no change” to “somewhat better” for the majority of participants (58%). 

### 3.4. Evaluation of the Videos

The majority of participants rated aspects of each of the videos highly. They reported high satisfaction and enjoyment, and stated that the information provided will have an impact on their practice (Supplementary File 3). 

### 3.5. Follow-Up Assessment 

At the 4–6 month follow-up, 54 HCPs (physicians (*n* = 23), nurses/nurse practitioners (*n* = 14), and registered dietitians (*n* = 17)) completed the survey, representing an 83% follow-up rate. Participant characteristics are presented in Table 2. In brief, the majority of participants were white females between the ages of 25–34 years, and the median number of years in practice was 8.5 (2.6–20.0). The majority had indicated at follow-up that they saw at least one pediatric patient with obesity a month (38.9%), and did not use the 5As approach to Pediatric Obesity Management (51.9%, Table 3).

### 3.6. Impact on Clinic Practice at Follow-Up

**Figure 4:**
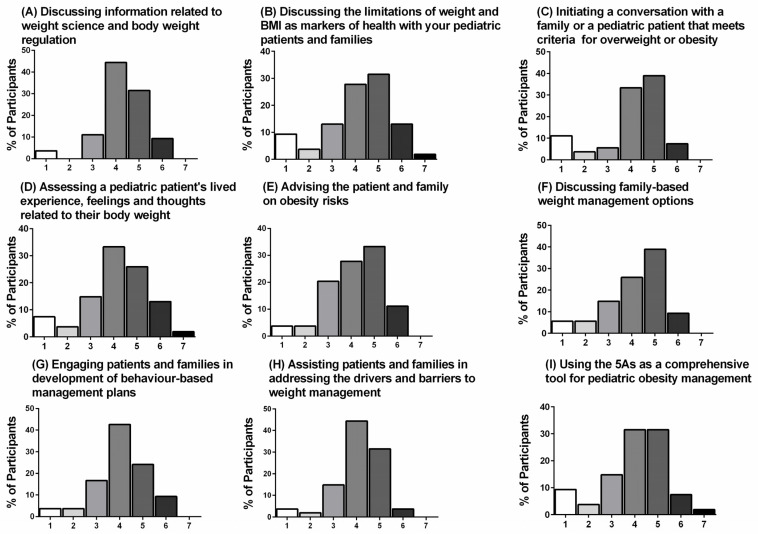
(**A**–**I**). Impact on practice 4 to 6 months later. Participant agreement (%) measured using a Likert-type scale (1 to 7, where 1 = No change, 2 = Hardly any change, 3 = No noticeable change, 4 = Somewhat changed, but the change has not made a big difference, 5 = A noticeable change, 6 = A real and worthwhile difference in my practice, and 7 = Made all the difference in my practice) is shown for each statement (*n* = 54).

When asked about practice change related to the topics discussed in the six videos, 26–44% of participants selected “Somewhat changed, but the change has not made a big difference”, while 24–39% of participants selected “A noticeable change.” The breakdown of participant rankings is presented in Figure 4A–I. 

### 3.7. Self-Efficacy Ratings from Baseline to Follow-Up

Self-efficacy ratings increased significantly from baseline to 4–6 months, for “discussing information related to weight science and body weight regulation” (Video 1; 54.7 ± 4.9 vs. 63.5 ± 4.2, *p* = 0.006), “assessing a pediatric patient’s lived experience, feelings and thoughts related to their body weight” (Video 4; 55.8 ± 7.0 vs. 64.6 ± 7.0, *p* = 0.008), “discussing family-based weight management options” (Video 5; 60.7 ± 6.3 vs. 67.1 ± 6.3, *p* = 0.026), “engaging patients and families in development of behavior-based management plans” (Video 5; 58.3 ± 5.9 vs. 66.2 ± 5.9, *p* = 0.013), “assisting patients and families in addressing the drivers and barriers to weight management” (Video 5; 55.0 ± 4.6 vs. 62.5 ± 4.1, *p* = 0.014), and “using the 5As as a comprehensive tool for pediatric obesity management” (Video 6; 32.8 ± 6.4 vs. 60.0 ± 6.4, *p* < 0.001). Self-efficacy ratings were not different over time (*p* > 0.05) for “discussing the limitations of weight and BMI as markers of health with your pediatric patients and families”, “initiating a conversation with a family or a pediatric patient that meets criteria for overweight or obesity”, or “advising the patient and family on obesity risks” (*p* > 0.05).

## 4. Discussion

In our study, HCPs’ ratings of perceived knowledge and self-efficacy improved after watching educational videos on how to address weight-related issues with pediatric patients and their families using the 5As framework. The toolkit was highly rated in terms of interest, ease of understanding, amount of information presented, impact on practice, learning, length, satisfaction, and enjoyment. Four to six months after viewing the toolkit, the majority of HCPs reported practice change attributable to the videos, and their self-efficacy ratings at follow-up significantly improved from baseline. These findings suggest that video-based education is one promising strategy to improve HCPs’ self-efficacy in addressing weight-related topics with children and their families.

Although baseline ratings for knowledge and self-efficacy were relatively high, it is notable that, with the exception of questions related specifically to the 5As, there was still a change in knowledge and self-efficacy immediately post-video. Baseline ratings for self-efficacy were higher than in previous studies [3,26], raising the possibility that HCPs’ confidence in treating pediatric patients with obesity may be improving, as previously suggested [33]. However, for certain aspects of care, such as “engaging patients and families in development of behavior-based management plans” and “assisting patients and families in addressing the drivers and barriers to weight management,” our findings indicate a need for further education and improvement. Indeed, the lowest ratings indicate a gap in knowledge about the 5As and in being confident using this framework when interacting with pediatric patients and their families. Post-video, we observed a change in self-reported behaviors, particularly for the questions related to the 5As. This improvement post-video is consistent with other studies employing video-based education [18,19,20,21], emphasizing the effectiveness of this type of resource for HCPs.

Post-video and 4–6 months following the intervention, the majority of HCPs described that the videos had led to practice change in regard to addressing weight-related issues. This finding of a positive impact on clinical practice using a video intervention has been previously described and supported [21], and extends the findings of our previous work [26]. Concordant with this, the majority of respondents rated the video content highly, raising the possibility that they would return to these resources at a later time if needed. The ability to use these resources for remote and asynchronous learning may lead to even greater reach and impact on practice.

Self-efficacy ratings increased from baseline to the 4–6 month follow up for a number of tasks related to addressing weight and using the 5As. This is similar to another study which found sustained confidence levels in dental students who watched an educational video in addition to standard teaching regarding the use of local anesthetics in children, as compared to students receiving standard teaching only [18]. However, self-efficacy did not improve for “discussing the limitations of BMI”, “advising on obesity risks”, and “initiating a conversation about weight”, a finding we also observed in our previous study [26]. At this time it is unclear why HCPs did not report an improvement over time. One possibility is that these questions probe subject material which is sensitive, and thus, may be difficult to convey to children and adolescents during a short clinic visit. It is likely that additional and perhaps varied resources are needed to broach these difficult topics.

There are a few limitations to be considered. First, our study sample is subject to selection bias due to the self-selected recruitment process and the likelihood that participating HCPs possess a strong interest in pediatric weight-related issues. Our study focused on physicians, nurses/nurse practitioners, and dietitians, and excluded other HCPs who work with pediatric patients with obesity, such as psychologists or physiotherapists, which may have altered the findings. Second, because the ethnic background of our HCPs was homogeneous, our findings do not necessarily reflect the diversity of HCPs in our community, and hence, we cannot fully account for differences in self-efficacy that may have been influenced by different ethnic or cultural backgrounds. Third, our study does not account for other training or knowledge that may have influenced self-efficacy. Future research should include an assessment of the broad range of medical education activities utilized by HCPs that might impact self-efficacy. Fourth, HCPs were not asked to self-report their body weight and body satisfaction, which may have implications for how they addressed weight with their patients. Lastly, HCPs self-rated their knowledge and self-efficacy, although perceived self-efficacy has been identified as an important determinant of behavior [34,35]. In terms of study strengths, our follow-up analysis allowed us to examine self-efficacy over time. Although the 4–6 month follow-up may have been relatively short, the finding that self-efficacy did not decline suggests that the information presented in the videos was retained. Including longer follow-up periods will help provide clarity on whether self-efficacy is maintained over an extended timeframe. Furthermore, using video-based educational resources is timely, and has the additional advantages of being readily consumable, easily translated into different languages, and efficient and cost-effective compared to traditional lectures or paper-based education [36]. It also allows for rapid dissemination and asynchronous access to materials and permits users to access resources remotely and use them at their own pace [36].

## 5. Conclusions

In summary, our evidence-informed toolkit of videos based on the 5As framework fills a considerable educational gap, led to changes in self-reported behaviors among HCPs, and sustained improvements in their self-efficacy in addressing weight-related topics with children and their families. Obesity is a complex disease and management should emphasize health over weight loss, begin early in life, and be family-centric. In order to promote healthy lifestyle behaviors and prevent eating disorders and other weight-related comorbidities, there is a critical need for tools to help HCPs engage in discussion around these topics in a sensitive, positive, and effective manner. Developing useful and effective resources for HCPs is a necessary component of medical education that has the potential to enhance client–provider interaction and clinical outcomes. In the future, this toolkit may be further evaluated with other HCPs (e.g., secondary and tertiary care physicians, physiotherapists, occupational therapists, psychologists, and social workers) to determine the impact of this resource across medical disciplines.

## Figures and Tables

**Table 1:** Description of videos in the toolkit.

Video	Description	
1. Introduction to Weight Science	This video presents background information, including the biologic processes involved in body weight regulation.	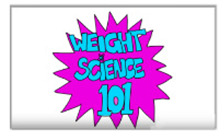
2. A Word About BMI	This video discusses how to use growth charts, as well as the limitations of weight and BMI as markers of health.	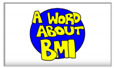
3. ASK	This video reviews how to approach discussions about obesity with pediatric patients and their caregivers.	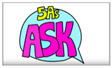
4. ASSESS	This video reviews how to comprehensively assess children and youth with obesity using The Edmonton Obesity Staging System for Pediatrics, which captures the severity of disease, as well as factors that complicate management, within four domains of health most commonly encountered in obesity—metabolic, mechanical, mental health, and social milieu (the 4Ms).	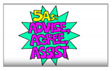
5. ADVISE, AGREE, and ASSIST	This video discusses the interventions for pediatric obesity management in the primary care setting, how to ensure patient engagement in the process, and how to sustain therapy.	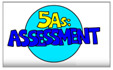
6. Putting it All Together	This video summarizes each of the 5As and how to use them as a comprehensive tool for pediatric obesity management, within a sample patient encounter.	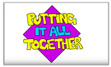

**Table 2:** Participant Characteristics.

	Baseline (*n* = 65)	Follow-Up (*n* = 54)
Age group (%)		
• <18 years	0	0
• 18–24 years	1.5	1.9
• 25–34 years	44.6	48.1
• 35–44 years	24.6	24.1
• 45–54 years old	7.7	3.7
• >55 years	20.0	20.4
I prefer not to answer	1.5	1.9
Sex (%)		
• Male	16.9	18.5
• Female	83.1	81.5
Ethnicity (%)		
• White	73.8	72.2
• Black	0	0
• Hispanic or Latino	3.1	3.7
• Aboriginal	1.5	1.9
• Asian or Pacific Islander	12.3	13.0
• South Asian or Middle Eastern	4.6	5.6
• I prefer not to answer	4.6	3.7
Number of years in practice ^1^	9.5 (2.9–20.0)	8.5 (2.6–20.0)

^1^ Data are presented as median [interquartile range].

**Table 3:** Questions about current practice and 5As.

	Baseline (*n* = 65)	Follow-Up (*n* = 54)
In your current practice, how often do you see children who are overweight or obese? (%)		
Never	0	0
Fewer than one patient every 12 months	4.6	3.7
At least one patient every 12 months	3.1	3.7
At least one patient every 6 months	10.8	22.2
At least one patient a month	32.3	38.9
At least one patient a week	36.9	18.5
At least one patient per day or more	12.3	13.0
In your current practice, do you use the 5As approach to Pediatric Obesity Management? (%)		
Yes	24.6	25.9
No	55.4	51.9
I don’t know	20.0	22.2
Have you received training on using the 5As of Pediatric Obesity Management? (%)		
Yes	16.9	
No	78.5	
I don’t know	4.6	
If yes, when did you receive this training? (%)		
During health professional school	27.3	
An academic/education meeting	54.5	
Other	18.2	
If yes, what type of training did you receive? (%)		
Didactive (Lecture format)	27.3	
Interactive	9.1	
E-learning	9.1	
Self-directed	18.2	
Combination	36.4	
If yes, how long was your training in total? (%)		
<1 h	54.5	
4 h	9.1	
1 day–1 week	36.4	
1 week	0	

**Table 4:** Baseline knowledge scores ^1^.

Question.	Baseline Rating (0–100)
Video 1: “Understanding information related to weight science and body weight regulation”	73.0 (50.0–80.0)
Video 2: “Understanding how to use BMI growth charts in your practice”	85.0 (65.5–95.0)
“Understanding the limitations of weight and BMI as markers of health”	76.0 (50.0–92.5)
Video 4: “Understanding obesity related comorbidities”	75.0 (60.0–85.0)
“Understanding the 4Ms framework”	41.0 (8.5–70.0)
“Understanding potential root causes and drivers of weight gain and barriers for weight management”	70.0 (50.0–80.0)
Video 6: “Knowledge about the 5As as a comprehensive tool for pediatric obesity management”	31.0 (6.5–53.0)

^1^ Knowledge was assessed by having the participant rate their knowledge for each question on a scale from 0 (not knowledgeable at all) to 100 (highly knowledgeable) (*n* = 65). Ratings are presented as median and interquartile ranges.

**Table 5:** Baseline self-efficacy scores ^1^.

Task	Baseline Rating (0–100)
Video 1: “Discussing information related to weight science and body weight regulation”	69.0 (50.0–80.0)
Video 2: “Using BMI growth charts in your practice”	90.0 (71.5–97.0)
“Discussing the limitations of weight and BMI as markers of health”	75.0 (50.0–87.5)
Video 3: “Initiating conversation with a family or a pediatric patient that meets criteria for overweight or obesity”	73.0 (50.0–90.0)
Video 4: “Assessing a pediatric patient’s lived experience, feelings, and thoughts related to their body weight”	65.0 (43.0–80.0)
“Using the 4Ms framework”	40.0 (11.0–67.5)
Video 5: “Advising the patient and family on obesity risks”	75.0 (64.2–83.7)
“Discussing family-based weight management options”	74.0 (50.0–84.5)
“Engaging patients and families in development of behavior-based management plans”	62.0 (50.0–81.5)
“Assisting patients and families in addressing the drivers and barriers to weight management”	60.0 (50.0–80.0)
Video 6: “Using the 5As as a comprehensive tool for pediatric obesity management”	31.0 (6.5–53.0)

^1^ Self-efficacy was assessed by having the participant rate their confidence performing the tasks on a scale from 0 (cannot do at all) to 100 (highly certain can do) (*n* = 65). Ratings are presented as median and interquartile ranges.

## Data Availability

The data that support the findings of this study are available from the corresponding author upon reasonable request.

## Supplementary Materials

The following are available online at https://www.mdpi.com/article/10.3390/ijerph18147568/s1.
