# Supplementary material for: Evaluation of a Pediatric Obesity Management Toolkit for Health Care Professionals: A Quasi-Experimental Study
Source: Int J Environ Res Public Health. 2021 Jul 16;18(14):7568. doi: 10.3390/ijerph18147568 (PMC8306158; doi:10.3390/ijerph18147568)
Supplement: Supplementary file 1 [file ijerph-18-07568-s001.zip › ijerph-1273494-supplementary.pdf]

Evaluation of the videos

For **Video 1**, participants rated the following video features as a 4 or 5 out of 5: "The content was presented in an interesting way that held my attention" (91%; 59 participants), "The information I received was easy to understand" (92%; 60 participants), "The information provided will impact my practice" (83%; 54 participants), "I would recommend this video to a colleague or trainee" (86%; 56 participants), "I learned something new" (83%; 54 participants), "The content of the video covered what I wanted to learn" (83%; 54 participants). 100% of participants thought the length of the video was appropriate and 98% felt they received the right amount of information. Satisfaction and enjoyment were both rated as 89% (4 or 5 out of 5).

For **Video 2**, participants rated the following video features as a 4 or 5 out of 5: "The content was presented in an interesting way that held my attention" (98%; 64 participants), "The information I received was easy to understand" (94%; 61 participants), "The information provided will impact my practice" (85%; 55 participants), "I would recommend this video to a colleague or trainee" (89%; 58 participants), "I learned something new" (75%; 49 participants), "The content of the video covered what I wanted to learn" (86%; 56 participants). 100% of participants thought the length of the video was appropriate and 92% felt they received the right amount of information. Satisfaction and enjoyment were both rated as 95% (4 or 5 out of 5).

For **Video 3**, participants rated the following video features as a 4 or 5 out of 5: "The content was presented in an interesting way that held my attention" (89%; 58 participants), "The information I received was easy to understand" (98%; 64 participants), "The information provided will impact my practice" (88%; 57 participants), "I would recommend this video to a colleague or trainee" (89%; 58 participants), "I learned something new" (77%; 50 participants), "The content of the video covered what I wanted to learn" (81%; 53 participants). 94% of

participants thought the length of the video was appropriate and 98% felt they received the right amount of information. Satisfaction and enjoyment were both rated as 85% (4 or 5 out of 5).

For **Video 4**, participants rated the following video features as a 4 or 5 out of 5: "The content was presented in an interesting way that held my attention" (94%; 61 participants), "The information I received was easy to understand" (95%; 62 participants), "The information provided will impact my practice" (85%; 55 participants), "I would recommend this video to a colleague or trainee" (92%; 60 participants), "I learned something new" (88%; 57 participants), "The content of the video covered what I wanted to learn" (89%; 58 participants). 94% of participants thought the length of the video was appropriate and 91% felt they received the right amount of information. Satisfaction and enjoyment were both rated as 90% (4 or 5 out of 5).

For **Video 5**, participants rated the following video features as a 4 or 5 out of 5: "The content was presented in an interesting way that held my attention" (95%; 62 participants), "The information I received was easy to understand" (95%; 62 participants), "The information provided will impact my practice" (83%; 54 participants), "I would recommend this video to a colleague or trainee" (92%; 60 participants), "I learned something new" (78%; 51 participants), "The content of the video covered what I wanted to learn" (85%; 55 participants). 97% of participants thought the length of the video was appropriate and 92% felt they received the right amount of information. Satisfaction and enjoyment were rated as 89 and 94%, respectively (4 or 5 out of 5).

For **Video 6**, participants rated the following video features as a 4 or 5 out of 5: "The content was presented in an interesting way that held my attention" (98%; 64 participants), "The information I received was easy to understand" (98%; 64 participants), "The information provided will impact my practice" (91%; 59 participants), "I would recommend this video to a

colleague or trainee” (94%; 61 participants), “I learned something new” (86%; 56 participants), “The content of the video covered what I wanted to learn” (92%; 60 participants). 98% of participants thought the length of the video was appropriate and 98% felt they received the right amount of information. Satisfaction and enjoyment were rated as 92 and 95%, respectively (4 or 5 out of 5).
